# Quality by Design-Based Development of a Stability-Indicating RP-HPLC Method for the Simultaneous Determination of Methylparaben, Propylparaben, Diethylamino Hydroxybenzoyl Hexyl Benzoate, and Octinoxate in Topical Pharmaceutical Formulation

**DOI:** 10.3797/scipharm.1312-20

**Published:** 2014-02-27

**Authors:** Chinmoy Roy, Jitamanyu Chakrabarty

**Affiliations:** ^1^ Analytical Research and Development, Integrated Product Development, Dr. Reddy’s Laboratories Ltd., Bachupally, Hyderabad-500090, Andhra Pradesh, India.; ^2^ Department of Chemistry, National Institute of Technology, Durgapur-713209, West Bengal, India.

**Keywords:** DHHB, Octinoxate, Degradation, RP-HPLC, DOE

## Abstract

A stability-indicating RP-HPLC method has been developed and validated for the simultaneous determination of methylparaben (MP), propylparaben (PP), diethylamino hydroxybenzoyl hexyl benzoate (DAHHB), and octinoxate (OCT) in topical pharmaceutical formulation. The desired chromatographic separation was achieved on the Kinetex^TM^ C18 (250 × 4.6 mm, 5 μm) column using gradient elution at 257 nm detection wavelength. The optimized mobile phase consisted of a buffer : acetonitrile : tetrahydrofuran (60 : 30 : 10, v/v/v) as solvent A and acetonitrile : tetrahydrofuran (70 : 30, v/v) as solvent B. The method showed linearity over the range of 0.19–148.4 μg/mL, 0.23–15.3 μg/mL, 1.97–600.5 μg/mL, and 1.85–451.5 μg/mL for MP, PP, DAHHB, and OCT, respectively. Recovery for all the components was found to be in the range of 98–102%. The stability-indicating capability of the developed method was established by analysing the forced degradation samples in which the spectral purity of MP, PP, DAHHB, and OCT, along with the separation of the degradation products from the analyte peaks, was achieved. The proposed method was successfully applied for the quantitative determination of MP, PP, DAHHB, and OCT in the lotion sample. The design expert with ANOVA software with the linear model was applied and a 2^4^ full factorial design was employed to estimate the model coefficients and also to check the robustness of the method. Results of the two-level full factorial design, 2^4^ with 20 runs including four centrepoint analysis based on the variance analysis (ANOVA), demonstrated that all four factors, as well as the interactions of resolution between DAHHB and OCT are statistically significant.

## Introduction

Direct exposure to UV radiation causes pronounced harmful effects on human health such as sunburn, hyperplasia, and immuneosuppression, also chronic responses including primarily photocarcinogenesis and photoaging [1].

Chemical sunscreens are generally aromatic compounds conjugated with an electron donating group and an electron acceptor group. This chemical structure favours electron delocalization and therefore helps the excitation of molecules from the ground state to an excited state. The energy required for this transition corresponds to the energies of ultraviolet A (UVA) and ultraviolet B (UVB) radiations [2, 3].

Chronic exposure to UVB (280–320 nm wavelengths) induces damage to human skin, such as burns and erythema. Volumes of evidence also demonstrate that UVA radiation (320–400 nm) contributes to photoaging, which results in the accumulation of massive amounts of abnormal elastic material in the dermis of photoaged skin and modifications in the collagen structure [2–6].

The necessity to provide high SPF (sun protection factor) and screening efficiency against both UVA and UVB wavelengths has led to the development of sunscreen formulations comprising multiple sunscreen chemicals [7].

**Fig. 1.**
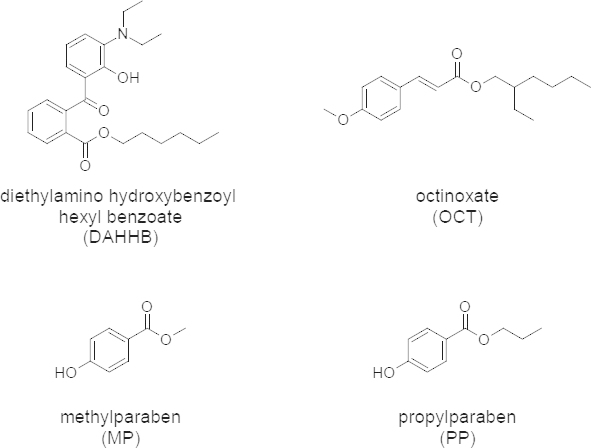
Chemical structure of DAHHB, OCT, MP, and PP.

Uvinul A plus, chemically known as diethylamino hydroxybenzoyl hexyl benzoate (DAHHB) (Fig. 1), is an oil-soluble UVA filter, which makes it highly desirable in a broad-spectrum sunscreen, used in sunscreen formulations at concentrations up to 10%, alone or in combination with other UV absorbers. Octinoxate, chemically known as *p*-Methoxycinnamic acid 2-ethylhexyl ester (OCT) (Fig. 1), is a UVB filter, frequently used in combination with other UVA absorbers to achieve high SPF values in the final product [8–11].

The preservative system is an important part of semisolid formulations for preventing the deterioration of formulations from microbial contamination. Methylparaben (Fig. 1) and propylparaben (Fig. 1) are the most commonly used preservatives and have been used for many years.

The literature survey reveals that several techniques have been reported such as derivative spectrophotometry [12], high-performance liquid chromatography (HPLC) [13–22], high-performance liquid chromatography–mass spectrometry (HPLC-MS) [23], *in vitro* assessment of skin penetration [24] for individual or in combination of sunscreen agents or in combination with preservatives in topical pharmaceutical formulation.

The combination of DAHHB and OCT is not available in any pharmacopoeia. So far, no reversed-phase liquid chromatography (RPLC) stability-indicating method has been reported for the rapid and simultaneous determination of MP, PP, DAHHB, and OCT in topical pharmaceutical formulation. Therefore, it is necessary to develop a new, rapid, and stability-indicating method for the simultaneous determination of four compounds (MP, PP, DAHHB, and OCT) in topical pharmaceutical formulation. The proposed method should be able to separate MP, PP, DAHHB, and OCT from each other and also from other degradation products. The design of experiment (DOE) technique was employed to study the effect of critical factors on the method performance. Furthermore, this method was validated according to the ICH guideline [25] and was successfully applied for the separation and quantification of all compounds of interest in the topical pharmaceutical formulation.

## Results and Discussion

### Method Development and Optimization

The primary target of developing the HPLC method is to achieve the simultaneous determination of MP, PP, DAHHB, and OCT in topical formulations under common chromatographic conditions; those that are applicable to the routine quality control of products in the pharmaceutical and cosmetic industries.

The optimizations of the column selection and mobile phase selection were done simultaneously. When stationary phases such as the Zorbax SB C18, Waters Symmetry

C18, and Inertsil ODS 3V were tried with mobile phases such as glacial acetic acid, triethylamine buffer (pH 2.5), and their combination with methanol, acetonitrile, and tetrahydrofuran, co-elution of the DAHHB peak and placebo peaks, peak broadening of OCT, and placebo peak interferences were observed. Good chromatography was observed using the Kinetex C-18 (250 × 4.6 mm, 5μm) column. A mixture of the buffer (phosphoric acid buffer pH 2.5 : acetonitrile : tetrahydrofuran (60 : 30 : 10, v/v/v) used as solvent A, and a mixture of acetonitrile : tetrahydrofuran (70 : 30, v/v) was used as solvent B. The wavelength was selected by injecting a known concentration of each of MP, PP, DAHHB, and OCT into the HPLC with a PDA detector and evaluating the UV spectra of each component. A common wavelength for the simultaneous determination of all components was selected as 257 nm by overlaying the spectra and wavelengths at which all components had significant absorbance. Other chromatographic parameters were optimized such as the flow rate of 1.2 ml/min, column temperature of 30°C, and injection volume (10 μL).

**Fig. 2.**
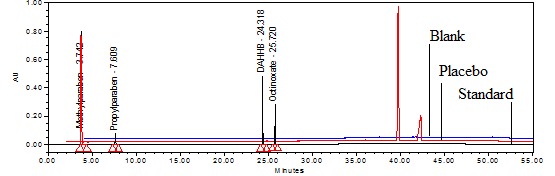
Typical overlay chromatogram of the blank, placebo, and standard preparation

Extraction of the active components from the semisolid sample matrix with acceptable recovery is a very critical aspect for sample preparation and was achieved by selecting the right diluent in the following manner. Considering the solubility of all the components, acetonitrile was used as diluent and satisfactory recovery was obtained. Based on the above experimental data, the chromatographic separation was finalized by the following gradient program time (min)/mobile phase A (%)/mobile phase B (%); 0.0/100/0, 9/100/0, 19/55/45, 31/15/85, 34/10/90, 44/10/90, 50/100/0, 55/100/0, at a flow rate of 1.2 mL/min at 30°C (column oven) temperature, detection wavelength 257 nm with 10 μL injection volume. By using the above chromatographic conditions and diluent, the standard, sample, and placebo preparation were made and injected into the HPLC with the developed parameters (Fig. 2).

### Experimental Design Approach

**Fig. 3.**
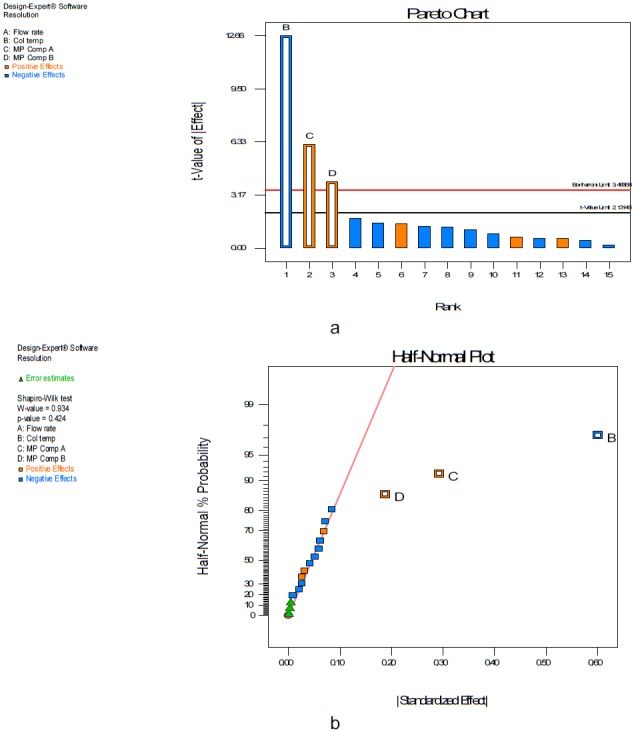
(a) Pareto chart and (b) half normal plot showing the effect of factor B > C > D in the resolution between the DAHHB and OCT peaks

**Fig. 4.**
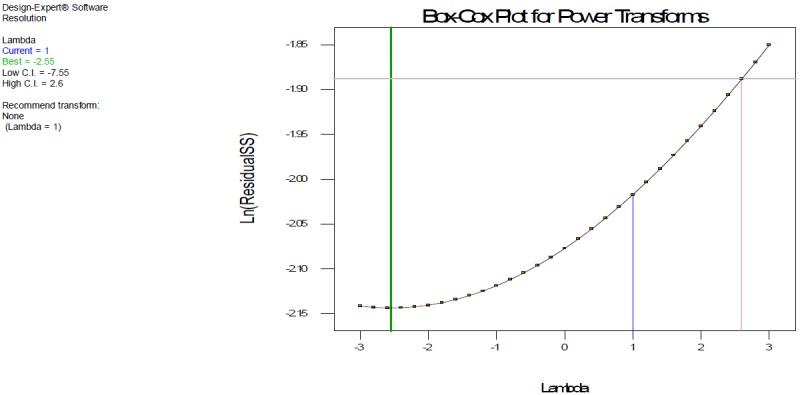
Box-Cox plot shows significance of the model (no power transformation required)

**Fig. 5.**
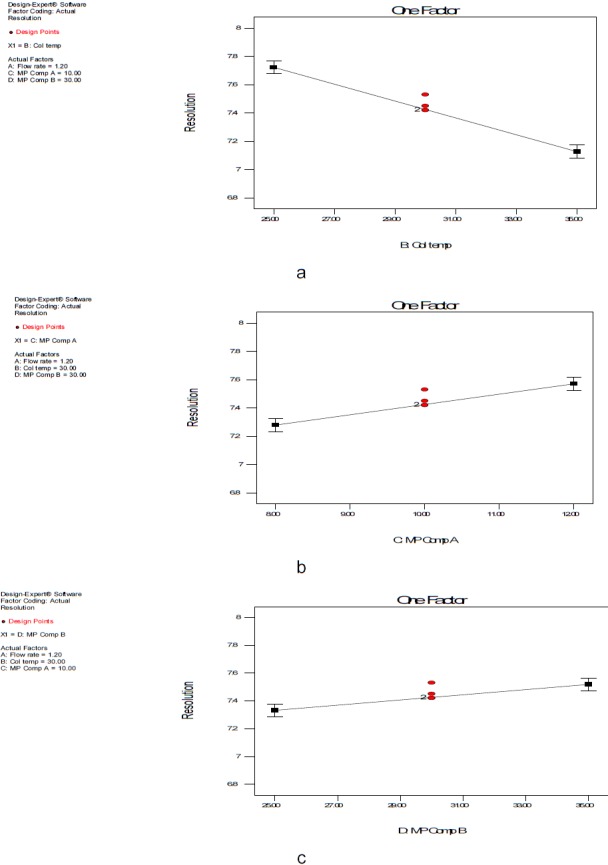
Single factor interaction in the resolution between the DAHHB and OCT peaks (a) column temperature (b) mobile phase A THF composition and (c) mobile phase B THF composition

Design Expert Software (Stat-Ease Inc., Statistics made easy, Minneapolis, MN, USA, version 7.0.0) was used for the experimental design throughout this screening process study. The full factorial design requires fewer measurements than the classical one-at-a-time experiment to give the same precision. At the same time, it detects and estimates any interaction between the factors. In order to study the simultaneous variation of the factors on the considered responses, a multivariate approach using design of experiments is recommended in robustness testing. However, if an analytical method is fast and requires the testing of a few factors (three or fewer), a good choice for robustness testing may be design expert, widely employed because of its high efficiency with respect to a fewer number of runs required in full factorial mode. In order to study the four variables at two levels, the design used in robustness testing of the resolution between the DAHHB and OCT peaks was a 2^4^ full factorial design. ANOVA with a linear model was applied to estimate the model coefficients and also check the robustness of the method. The results of the two-level full factorial design, 2^4^ with 20 runs, includes four centrepoints. The effects of the three factors in the resolution between the DAHHB and OCT peaks are shown in a Pareto chart (Figure 3(a)) half normal plot (Figure 3(b)). Four factors were considered: flow rate mL/min (A), column temperature (B), tetrahydrofuran composition% in mobile phase A (C), and tetrahydrofuran composition% in mobile phase B (D). The factors and level considered (response) for the studies are shown in Table 1. Standards of DAHHB and OCT were prepared in assay concentrations. The critical resolution between the DAHHB and OCT peaks was studied as a response. No power transformation for the significance of the model was required as shown in the Box-Cox plot (Figure 4). The effect plot of the different factors revealed a decrease in the resolution between the DAHHB and OCT peak by an increase in factor B (column temperature) (Figure 5(a)), while an increase in the resolution between the DAHHB and OCT peak was observed with an increase in factor C (THF composition in mobile phase A) (Figure 5(b)), and factor D (THF composition in mobile phase B) (Figure 5(c)).

The ANOVA statistical test was employed to determine the significant and most contributing factors where they were ranked on the basis of the degree of *F*-ratio. The higher the *F*-value corresponds with the smaller “Prob>*F*” value, the more significant the resultant model and individual coefficient are. Table 2 shows the reading of the ANOVA analysis where the *F*-value and P-value of the model were 73.77 and 0.0001, respectively, demonstrating that the estimated model fits the experimental data satisfactorily.

**Fig. 6.**
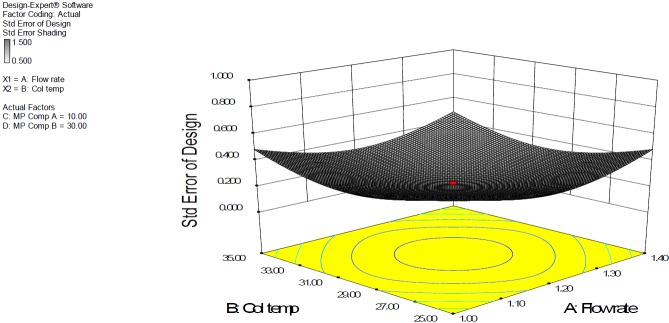
Three-dimensional plot of the full factorial for the predicted response resolution between the DAHHB and OCT peaks plotted on the y–axis as a function of factor A (flow rate) and B (column temperature); fixed factor: C (mobile phase A % tetrahydrofuran 10.0%) and D (mobile phase B % tetrahydrofuran 30.0%)

R^2^ refers to the proportion of the variation in the dependent variable accounted for by the independent variable. When R^2^ equals 1, the relationship is perfect and all values of the dependent and independent variables lie on a straight line. Adjusted R^2^ is the change of R^2^ that adjusts the number of terms in a model. It calculates the proportion of the variation in the dependent variable accounted by the explanatory variables. R^2^ always increases when a new term is added to a model, but adjusted R^2^ increases only if the new term improves the model. The adjusted R^2^ can be negative, and will always be less than or equal to R^2^. The predicted R^2^ indicates how well a regression model predicts responses for new observations. This statistic helps to determine when the model fits the original data but is less capable of providing valid predictions for new observations. Like adjusted R^2^, predicted R^2^ can be negative and it is always lower than R^2^. Table 3 shows the R^2^ value (0.9), which indicates that 90 % of the data variability was successfully explained by the model. This means that with a slight change in temperature, flow rate, and % tetrahydrofuran composition in the mobile phase during analysis, the resolution between the DAHHB and OCT peaks will not be affected. Hence, from this data generated by the model, it can be expounded that the resolution between the DAHHB and OCT peaks decreases with an increase in column temperature and increases with increasing % tetrahydrofuran composition in the mobile phase as shown in Figure 6.

**Tab. 1.** The range and levels of the variables in the 2^4^ full factorial design

Std.	Run	Factor 1 A: flow rate (mLmin^-1^)	Factor 2 B: column temp(°C)	Factor 3 C: THF comp (A%)	Factor 4 D: THF comp (B%)	Response 1 Resolution
5	1	1.0	25	12.0	25.0	7.73
14	2	1.4	25	12.0	35.0	8.09
2	3	1.4	25	8.0	25.0	7.52
3	4	1.0	35	8.0	25.0	6.93
17	5	1.2	30	10.0	30.0	7.45
12	6	1.4	35	8.0	35.0	7.04
9	7	1.0	25	8.0	35.0	7.68
18	8	1.2	30	10.0	30.0	7.53
8	9	1.4	35	12.0	25.0	7.02
15	10	1.0	35	12.0	35.0	7.37
11	11	1.0	35	8.0	35.0	7.15
1	12	1.0	25	8.0	25.0	7.40
19	13	1.2	30	10.0	30.0	7.42
13	14	1.0	25	12.0	35.0	8.01
16	15	1.4	35	12.0	35.0	7.21
10	16	1.4	25	8.0	35.0	7.54
7	17	1.0	35	12.0	25.0	7.32
4	18	1.4	35.0	8.0	25.0	6.92
6	19	1.4	25.0	12.0	25.0	7.76
20	20	1.2	30.0	10.0	30.0	7.42

**Tab. 2.** ANOVA for 2^4^ full factorial design: Response: Resolution between DAHHB and OCT peaks

Source	Sum of Squares	df	Mean Square	F Value	p-value Prob > F	Remarks
Model	1.90	3	0.63	73.77	< 0.0001	significant
B-Column Temp.	1.42	1	1.42	165.62	< 0.0001	
C-MP comp. A	0.34	1	0.34	39.52	< 0.0001	
D- MP comp.B	0.14	1	0.14	16.16	0.0010	
Residual	0.14	16	8.586E-003			
Lack of Fit	0.13	13	9.944E-003	3.68	0.1552 n	not significant
Pure Error	8.100E-003	3				
Cor Total	2.04	19				

Df: degree of freedom, Cor Total: corrected total sum of squares, Values of Prob> F less than 0.05 indicates models are statistically significant

**Tab. 3.** The regression equation obtained for resolution for coded factors, along with the regression parameters are tabulated below:

Response	Regression equation for coded factors	R2	Predicted R^2^	Adjusted R^2^
Resolution	+7.43 - 0.30*B + 0.15*C + 0.093*D	0.9326	0.8875	0.9199

### Design Space

**Fig. 7.**
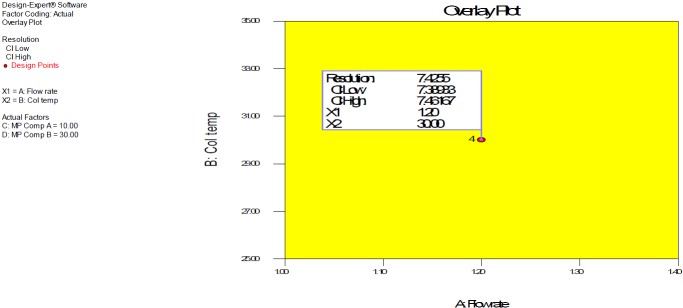
Graphical plot of the full factorial design space for between two factors A (flow rate) and B (column temperature) at fixed factor C (THF composition in mobile phase A) and factor D (THF composition in mobile phase B)

DOE revolves around the concept of the design space, the multidimensional combination and interaction of input variables and process parameters that have demonstrated to provide the assurance of quality. Working within the design space is not considered as a change. Movement out of the design space is considered to be a change and would normally initiate a regulatory post-approval change process. Design space is proposed by the applicant and is subject to regulatory assessment and approval [27–30]. Design space was established by employing full factorial design variables of the flow rate (A) 1.0–1.4 mL/ min, column temperature (B) 25–35°C, % tetrahydrofuran composition in mobile phase A (C) 8–12%, and % tetrahydrofuran composition in mobile phase B (C) 25–35% and their respective responses which are presented in Figure 7.

### Analytical Method Validation

After satisfactory development of the method, it was subjected to method validation as per ICH guidelines [25, 26]. The method was validated to demonstrate that it is suitable for its intended purpose by the standard procedure to evaluate adequate validation characteristics (system suitability, accuracy, precision, linearity, limit of detection, limit of quantification, robustness, solution stability, filter compatibility, and stability-indicating capability).

### System Suitability

System suitability parameters were measured so as to verify the system, method, and column performance. The system precision was determined by five replicate injections of the standard preparation. Results of the system suitability parameters such as % RSD (related standard deviation), theoretical plates, and tailing factor are presented in Table 4.

### Method Precision (Repeatability)

The precision of the assay method was evaluated by carrying out six independent determinations of 100 μg/mL of MP, 10 μg/mL of PP, 400 μg/mL of DAHHB, and 300 μg/mL of OCT in lotion samples against qualified working standards. The average % assay (n=6) of MP, PP, DAHHB, and OCT were 101.0%, 101.3%, 101.0%, and 100.9%, respectively, with the RSD below 0.5%. Low values of % RSD indicate that the method is precise (Table 5).

### Intermediate Precision (Reproducibility)

The purpose of this study was to demonstrate the reliability of the test results with variations. The reproducibility was checked by analyzing the samples by a different analyst using a different chromatographic system and column on a different day. Results are presented in Table 5.

**Tab. 4.** System suitability results (precision, intermediate precisions, robustness) for MP, PP, DAHHB, and OCT

Parameter	MP			PP			DAHHB			OCT		
	N^p^ >	T^F^ ≤	R^s^ ≤	N^p^ >	T^F^ ≤	R^s^ ≤	N^p^ >	T^F^ ≤	R^s^ ≤	N^p^ >	T^F^ ≤	R^s^ ≤
2000	2.0	2.0	2000	2.0	2.0	2000	2.0	2.0	2000	2.0	2.0
Precision	8746	1.2	0.3	15129	1.1	0.3	425964	1.0	0.3	442613	1.0	0.2
Intermediate Precision	7118	1.1	0.3	11306	1.0	0.2	249210	1.0	0.2	257856	1.0	0.2
At 1.4 mL/min flow rate	7736	0.9	0.2	11442	1.1	0.3	422154	1.0	0.3	459886	1.0	0.1
At 1.0 mL/min flow rate	9264	1.0	0.1	15733	1.1	0.1	441567	1.0	0.1	436456	1.0	0.1
At 35°C column temp.	8999	1.2	0.2	15511	1.1	0.6	446644	1.0	0.1	461604	1.0	0.1
At 35°C column temp.	8908	1.2	0.0	15274	1.1	0.1	417057	1.0	0.1	434903	1.0	0.0
Buffer pH 2.7	8897	1.2	0.1	15642	1.1	0.1	443340	1.0	0.1	459741	1.0	0.1
Buffer pH 2.3	8886	1.2	0.1	15761	1.1	0.2	442889	1.0	0.3	461218	1.0	0.3
Mobile phase A (THF +10 %)	7209	1.2	0.5	14605	1.1	0.2	376311	1.0	0.4	398632	1.0	0.4
Mobile phase A(THF –10 %)	7487	1.2	0.8	15166	1.1	0.9	392568	1.0	0.7	424632	1.0	0.6
Mobile phase B (THF +10 %)	8169	0.9	0.6	15679	1.0	0.9	459868	1.0	0.6	469536	1.0	0.5
Mobile phase B (THF –10 %)	7865	0.9	0.1	15191	1.0	0.3	453016	1.0	0.4	463408	1.0	0.3

THF Tetrahydrofuran; N^P^ Theroetical plates; T^F^ Tailing factor; R^S^ % RSD of five standards.

### Specificity

Specificity is the ability of the method to measure the analyte response in the presence of its potential impurities and placebo matrix [26]. Forced degradation studies were performed to demonstrate the selectivity and stability-indicating capability of the proposed RP-LC method. Figure 2 shows that there is no interference at the retention time of MP, PP, DAHHB, and OCT due to the blank or placebo. Overlay chromatograms of the blank, placebo, and standard are presented in Figure 2.

### Forced Degradation Studies

Forced degradation studies of the drug product were also performed to evaluate the stability-indicating property and specificity of the proposed method. Stress studies were performed at the concentrations of 100 μg/mL of MP, 10 μg/mL of PP, 400 μg/mL of DAHHB, and 300 μg/mL of OCT on the lotion formulation. The peak purity test was carried out for the MP, PP, DAHHB, and OCT peaks by using a PDA detector on the stress samples. All the solutions used in the forced degradation studies were prepared by dissolving the drug product in a small volume of diluent and further stressing agents. After degradation, these solutions were diluted with diluent to yield the stated MP, PP, DAHHB, and OCT concentrations of 100 μg/mL, 10 μg/mL, 400 μg/mL, and 300 μg/mL, respectively.

**Tab. 5.** Method precision and intermediate precision results, LOD, LOQ evaluations, and linearity data

Parameter	MP	PP	DAHHB	OCT
Precision (n=6) (% Assay ±	101.0 ± 0.15;	101.3 ± 0.46;	101.0 ± 0.57;	100.9 ± 0.51;
SD; % RSD; 95 % C.L.)	0.15; 0.12	0.45; 0.36	0.57; 0.46	0.50; 0.41
Intermediate-precision (n=6) (% Assay ± SD; % RSD; 95 % C.L.)	101.3 ± 0.53; 0.53; 0.43	101.7 ± 0.68; 0.68; 0.58	101.0 ± 0.95; 0.94; 0.76	100.8 ± 0.95; 0.94; 0.76
LOD (μg/mL)	0.057	0.14	0.592	0.555
LOQ (μg/mL)	0.19	0.46	1.97	1.85
Linearity-range (μg/mL)	0.19–148.4	0.23–15.3	1.97–600.5	1.85–451.5
Correlation coefficient	0.9999	0.9999	0.9999	0.9999
Intercept (a)	80690.184	296.964	2933.164	9034.352
Slope (b)	46553.021	41097.861	6387.574	4742.508
Bias at 100 % response	1.71	0.07	0.11	0.63

95% C.L. = 95% Confidence interval.

#### Acid Hydrolysis

Acidic degradation was carried out by adding 2 mL of 5 N HCl, and treating at 70°C in a water bath, and after 3 h the mixture was neutralized by adding 2 mL 5 N NaOH. In Fig. 7(b), significant degradation was observed for MP and one major degradation peak was observed at 2.5 min. Degradation was also observed for OCT with a degradation peak at 4.704 min. All the major and minor degradation products were well-separated from the MP, PP, DAHHB, and OCT peaks. The peak purity was checked for all four analytes and the results are summarized in Table 6.

#### Base Hydrolysis

Basic degradation was carried out by adding 1 mL of 5 N NaOH, and after 30 minutes the mixture was neutralized by adding 1 mL 5 N HCl. In Fig. 7(a), significant degradation was observed for MP, PP, and DAHHB, major degradation peaks were observed at 2.528, 4.719 min. All the degradation products were well-separated from the MP, PP, DAHHB, and OCT peaks. The peak purity was checked for all four analytes and the results are summarized in Table 6.

#### Hydrogen Peroxide Oxidation

Peroxide oxidation was carried out by adding 2 mL of 30% v/v H_2_O_2_, at 70°C for 1 h. Fig. 7 (c) shows significant degradation for OCT when the lotion sample was subjected to peroxide oxidation. All the degradation products were well-separated from the MP, PP, DAHHB, and OCT peaks. The peak purity was checked for all four analytes and the results are summarized in Table 6.

#### Thermal Degradation

The lotion sample and placebo sample were exposed to dry heat at 75°C for 6 hr. No degradation was observed for the thermally exposed samples (75°C, 6hrs). The peak purity was checked for all four analytes and the results are summarized in Table 6.

#### Photolytic Degradation

The lotion sample and placebo samples were exposed to visible light for 240 h resulting in an overall illustration of 1.2 million lux h, and UV light for 250 h resulted in an overall illustration of 200 w h/m^2^ at 25°C. No degradation was observed for photolytic degradation except PA. The peak purity was checked for all four analytes and the results are summarized in Table 6.

The purity and assay of MP, PP, DAHHB, and OCT were unaffected by the presence of its degradation products, thus confirming the stability-indicating power of the developed method.

**Tab. 6.** Results of forced degradation study for MP, PP, DAHHB, and OCT

Component		Acidic hydrolysis (5 N HCl, 70°C,3 h)	Alkaline hydrolysis (5 N NaOH, 70°C, 30 mins)	Peroxide oxidation (30% H_2_O_2_, 70°C, 1 h)	Thermal exposed (At 75°C, 6 h)	Photolytic exposed (1.2 million lux h and 200 wh/m^2^)
MP	%Deg.	3.0	15.4	ND	ND	ND
	PA	0.306	0.115	0.127	0.206	0.317
	PTH	1.099	1.305	1.569	1.354	1.492
PP	%Deg.	ND	3.3	ND	ND	5.3
	PA	2.710	2.180	2.668	4.182	3.458
	PTH	13.449	12.100	18.132	19.566	14.297
DAHHB	%Deg.	ND	3.1	ND	ND	ND
	PA	2.182	2.759	2.021	2.462	2.250
	PTH	3.257	4.146	4.299	4.707	4.149
OCT	%Deg.	2.9	ND	3.0	ND	ND
	PA	0.367	0.392	0.318	0.375	0.388
	PTH	1.764	1.313	1.144	1.267	1.341

N.D. No Degradation PA Purity angle PTH Purity Threshold

**Fig. 8.**
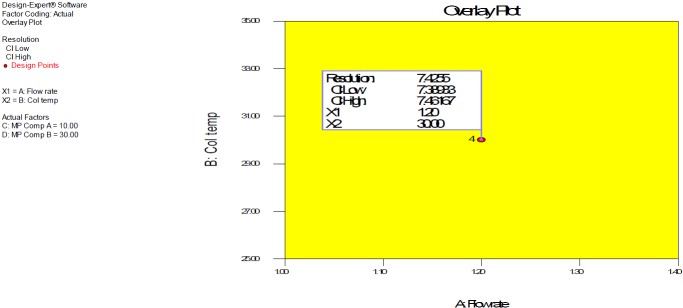
A typical chromatogram of (a) base hydrolysis sample, (b) acid hydrolysis sample, (c) peroxide oxidation sample

### Accuracy

The accuracy of an analytical method is the closeness of the test results obtained by that method compared to the true values. To confirm the accuracy of the proposed method, recovery experiments were carried out by the standard addition technique. Three different concentration levels (50%, 100%, and 150%) of the standards were added to pre-analyzed placebo samples in triplicate. The percentage recoveries of MP, PP, DAHHB, and OCT at each level and each replicate were determined. The mean of the percentage recoveries (n =3) and the % RSD (related standard deviation) were calculated. The amount recovered was within ±1% of the amount added, which indicates that the method is accurate and also there is no interference due to excipients present in the lotion sample. The results of the recoveries for the assay are shown in Table 7.

**Tab. 7.** Accuracy results

Components		At 50%	At 100%	At 150%
**MP**	% Recovery ^#^	100.0	98.8	98.5
	% R.S.D.^*^	0.15	0.15	0.33
**PP**	% Recovery ^#^	99.3	98.7	99.9
	% R.S.D.^*^	0.23	0.12	0.38
**DAHHB**	% Recovery ^#^	99.4	98.8	99.8
	% R.S.D.^*^	0.06	0.18	0.32
**OCT**	% Recovery ^#^	99.6	98.6	99.5
	% R.S.D.^*^	0.06	0.12	0.32

^*^ Determined on three values;

^#^ Mean of three determinations.

### Limit of Detection (LOD) and Quantification (LOQ)

The LOD and LOQ were determined at a signal-to-noise ratio of 3:1 and 10:1, respectively, by injecting a series of dilute solutions with known concentrations. The limit of detection and limit of quantification values of MP, PP, DAHHB, and OCT are reported in Table 5.

### Linearity

The linearity of an analytical method is its ability to elicit test results that are directly, or by a well-defined mathematical transformation, proportional to the concentration of the analyte. Linearity was demonstrated from the LOQ % to 150% of the standard concentration using a minimum of six calibration levels of the test concentration (LOQ-148.4 μg/mL for MP, LOQ-15.3 μg/mL for PP, LOQ-600.5 μg/mL for DAHHB, and LOQ-451.5 μg/mL for OCT), which gave us good confidence on the analytical method with respect to the linear range. The response was found to be linear for all MP, PP, DAHHB, and OCT from the LOQ to 150% of the standard concentration, and the correlation coefficient was also found to be greater than 0.9999. Bias was also found within ± 2.0. The result of the correlation coefficients, Y-intercept of the calibration curve, and % bias at 100 % response for MP, PP, DAHHB, and OCT are presented in Table 5.

### Robustness

The robustness as a measure of the method’s capacity to remain unaffected by small, but deliberate changes in chromatographic conditions was studied by testing the influence of small changes in flow rate (1.2 ± 0.2 mL/min), change in the column oven temperature (30 ± 5°C), mobile phase buffer pH (2.5 ± 2), mobile phase A tetrahydrofuran composition (10 ± 10%), and change in mobile phase B tetrahydrofuran composition (30 ± 10%). The system suitability parameters such as theoretical plates, tailing factor, and % RSD of MP, PP, DAHHB, and OCT standard were studied. In all of the deliberate varied chromatographic conditions, the system suitability parameters met the acceptance criteria. Thus, the method was found to be robust with respect to variability in the applied conditions. The results are presented in Table 4 along with the system suitability parameters of precision and the intermediate precision study. The resolution between the DAHHB and OCT peak was observed to be more than 6.5 for the robustness parameters. Thus, the method was found to be robust with respect to variability in the above conditions.

### Stability of Analytical Solutions

The stability of a sample solution was established by the storage of a sample solution at ambient temperature for 24 h. The lotion sample solution was re-analyzed after 12 and 24 h time intervals, and the assay was determined and compared against freshly prepared standard solutions. The variability in the assay of all four substances was within ± 1% during solution stability. The results from the solution stability experiments confirmed that the sample solution was stable for up to 24 h during the assay determination which are presented in Table 8.

**Tab. 8.** Solution stability results

% Assay	Initial	After 12 hrs.	After 24 hrs.
**MP**	100.7	100.7	100.8
**PP**	100.6	100.2	100.7
**DAHHB**	101.5	101.7	99.9
**OCT**	101.6	102.4	99.9

### Filter Compatibility

Filter compatibility was performed for the nylon 0.45 μm syringe filter (Millipore) and PTFE 0.45 μm syringe filter (Millipore). To confirm the filter compatibility in the proposed method, the filtration recovery experiment was carried out by a sample filtration technique. The sample was filtered through both syringe filters and the percentage assay was determined and compared against the centrifuged sample. The sample solution did not show any significant changes in the assay percentage with respect to the centrifuged sample. Percentage assay results are presented in Table 9. In the displayed results, the difference in % assay was not observed to be more than ±1.0, which indicates that both syringe filters have a good compatibility with the sample solution.

**Tab. 9.** Filter compatibility results

% Assay	Centrifuged Sample	Nylon filter 0.45μm	PTFE filter 0.45μm
**MP**	100.8	99.2	101.3
**PP**	101.5	100.1	102.4
**DAHHB**	101.6	100.6	101.2
**OCT**	102.0	101.0	101.7

## Experimental

### Chemicals, Reagents, and Samples

The lotion sample, placebo matrix, and working standards were provided by Dr. Reddys Lab, India. HPLC grade acetonitrile and orthophosphoric acid were used (Rankem, Delhi, India). Nylon membrane filters (0.45 μm), PTFE syringe filters (0.45 μm), and nylon syringe filters (0.45 μm) were from Millipore, Mumbai, India. Water for HPLC was generated using the Milli-Q Plus water purification system (Millipore, Milford, MA, USA).

### Equipment

The chromatographic analysis was performed using HPLC (Waters 2695 Alliance Separation Module) (Waters Milford, USA) equipped with a PDA detector, quaternary solvent manager, and autosampler system. The output signals were monitored and processed using Empower 2 software. The Cintex digital water bath was used for the hydrolysis studies. Photostability studies were carried out in the photostability chamber (SUN TEST XLS+, Atlas, USA). Thermal stability studies were performed in a dry air oven (Cintex, Mumbai, India).

### Chromatographic Conditions

All chromatographic experiments were performed using the Waters Kinetex^TM^ C18 (250×4.6 mm, 5μm) column. The optimized mobile phase consisted of a mixture of a buffer (phosphoric acid buffer pH 2.5 : acetonitrile : tetrahydrofuran (60 : 30 : 10, v/v/v) used as solvent A, and a mixture of acetonitrile : tetrahydrofuran (70 : 30, v/v) was used as solvent B. The mobile phase buffer was filtered through 0.45 urn nylon membrane filter and degassed under vacuum prior to use. The separation of MP, PP, DAHHB, OCT, and all impurities was achieved by gradient elution using solvent A and solvent B. Acetonitrile was used as diluent. A gradient program was used as time (min)/mobile phase A (%)/mobile phase B (%); 0.0/100/0, 9/100/0, 19/55/45, 31/15/85, 34/10/90, 44/10/90, 50/100/0, 55/100/0, at a flow rate 1.2 mL/min at 30°C. The detection wavelength was 257 nm.

### Standard Solution Preparation

The stock solutions of MP (1000 μg/mL), PP (1000 μg/mL), DAHHB (2000 μg/mL), and OCT (1500 μg/mL) were prepared by dissolving an appropriate amount of standard substances in acetonitrile, separately. Working standard solution was prepared by mixing the above stock solutions of MP, PP, DAHHB, and OCT with the final concentration of 100 μg/mL, 10 μg/mL, 400 μg/mL, and 300 μg/mL, respectively.

### Sample Solution Preparation

An accurately weighed 2.5 g sample (equivalent to 100 mg of DAHHB, 75 mg of OCT) was taken into a 50-mL volumetric flask. About 35 mL of acetonitrile was added to this volumetric flask and sonicated in an ultrasonic bath for 20 min with intermittent shaking, diluted to the volume with acetonitrile and mixed well. A portion of the solution was centrifuged at 3500 rpm for 15 minutes, supernatant solution was filtered through a 0.45 μm nylon syringe filter, and injected into the HPLC (used for quantification of MP and PP). Five-mL of the above supernatant solution was pipetted into a 25-mL volumetric flask, made up to the volume with diluent, and mixed well. A portion of solution was filtered with a 0.45 μm nylon filter and the filtrate was collected after discarding the first few milliliters (used for quantification of DAHHB and OCT).

### Placebo (Other Substances without MP, PP, DAHHB, and OCT) Solution Preparation

An accurately weighed 2.5 g of the placebo sample was taken into a 50-mL volumetric flask. About 35 mL of acetonitrile was added to this volumetric flask and sonicated in an ultrasonic bath for 20 min with intermittent shaking, diluted to the volume with acetonitrile, and mixed well. A portion of the solution was centrifuged at 3500 rpm for 15 minutes. Then we filtered the supernatant solution through 0.45 μm nylon syringe filter and injected it into the HPLC.

## Conclusion

The experimental design describes the key HPLC method components including column temperature, mobile phase flow rate, and % tetrahydrofuran composition in the mobile phase. The interrelationships were studied and the preliminary optimized conditions were obtained for each combination. Here, a better understanding of the factors influencing chromatographic separation and greater confidence in the ability of the methods to meet their intended purposes is done. Moreover, this approach ensures a better design of the product. A gradient RP-HPLC method was successfully developed for the simultaneous determination of methylparaben, propylparaben, diethylamino hydroxybenzoyl hexyl benzoate, and octinoxate in topical pharmaceutical formulation. The method validation results have proven that the method is selective, precise, accurate, linear, robust, filter compatible, and stability-indicating. Forced degradation data proved that the method is specific for the analytes and free from the interference of the placebo and degradation products. Moreover, it may be applied for the individual and simultaneous determination of methylparaben, propylparaben, diethylamino hydroxybenzoyl hexyl benzoate, and octinoxate in the study of content uniformity, tube homogeneity, and *in vitro* release test profiling of diethylamino hydroxybenzoyl hexyl benzoate and octinoxate topical pharmaceutical dosage forms, where the sample load is higher and high throughput is essential for faster delivery of results.
